# Eighteen-month-old infants represent nonlocal syntactic dependencies

**DOI:** 10.1073/pnas.2026469118

**Published:** 2021-10-04

**Authors:** Laurel Perkins, Jeffrey Lidz

**Affiliations:** ^a^Department of Linguistics, University of California, Los Angeles, CA 90095;; ^b^Department of Linguistics, University of Maryland, College Park, MD 20742

**Keywords:** syntax, language acquisition, nonlocal dependencies, *wh*-questions

## Abstract

The human ability to produce and understand an indefinite number of sentences is driven by syntax, a cognitive system that can combine a finite number of primitive linguistic elements to build arbitrarily complex expressions. The expressive power of syntax comes in part from its ability to encode potentially unbounded dependencies over abstract structural configurations. How does such a system develop in human minds? We show that 18-mo-old infants are capable of representing abstract nonlocal dependencies, suggesting that a core property of syntax emerges early in development. Our test case is English *wh*-questions, in which a fronted *wh*-phrase can act as the argument of a verb at a distance (e.g., *What did the chef burn?*). Whereas prior work has focused on infants’ interpretations of these questions, we introduce a test to probe their underlying syntactic representations, independent of meaning. We ask when infants know that an object *wh*-phrase and a local object of a verb cannot co-occur because they both express the same argument relation (e.g., *What did the chef burn the pizza). We find that 1) 18 mo olds demonstrate awareness of this complementary distribution pattern and thus represent the nonlocal grammatical dependency between the *wh*-phrase and the verb, but 2) younger infants do not. These results suggest that the second year of life is a period of active syntactic development, during which the computational capacities for representing nonlocal syntactic dependencies become evident.

## Significance

As mature speakers of a language, we are able to produce and understand an indefinite number of sentences. This ability comes from a powerful cognitive system—syntax—whose properties reveal the types of computations that human minds can engage in. One core property is the capacity to encode abstract grammatical dependencies that can hold at a distance. When does this property emerge in development? We identify a nonlocal dependency that 18-mo-old infants, but not younger infants, represent syntactically. These findings suggest that the second year of life is a period of active syntactic development and that even before they regularly produce full sentences of their own, infants demonstrate some of the core computational capacities that syntax relies on.

The human capacity for language is underwritten by a mentally represented formal system that allows us to produce and understand an indefinite number of sentences. Studying this formal system gives us a window into the kinds of computations a human mind can engage in and consequently what kinds of structures are native to the mind (1–4).

The computational centerpiece of the human linguistic capacity is syntax. It is syntax that gives us the capacity to combine a finite number of linguistic forms in order to express an infinite number of meanings. Natural language syntax does this in particular ways: it combines linguistic elements in hierarchical, potentially recursive structures and encodes abstract dependencies over these structures. For instance, the dependency between a verb and its direct object holds regardless of the particular verb or the particular object (underlined):

i. a. The chef burned the pizza.
b. The runner kicked the can.
c. The employee painted the blue fence surrounding the store.

In these sentences, the structural relation between the verb *burn* and its object *the pizza* is the same as the relation between *kick* and *the can* or between *paint* and *the blue fence surrounding the store*.

Whereas in a basic declarative clause, this relation requires adjacency in English (*ii*), in certain constructions, these relations can be established at a distance (*iii*).

ii. a. The chef burned (*entirely) the pizza.
b. The runner kicked (*very hard) the can.
c. The employee painted (*sloppily) the blue fence surrounding the store.
iii. a. What did the chef burn?
b. What did the manager say that the chef burned?
c. What did the manager who the customer called say that the chef burned?

Even though the fronted *wh*-phrase (*what*) can occur quite far away from *burn* in *iii*, it bears the same object relation to the verb as the corresponding phrase (*the pizza*) in *i*. The object *wh*-phrase and the direct object noun phrase (NP) can never co-occur (*iv*), suggesting that they are both expressions of the same argument relation.

iv. *What did the chef burn the pizza?

This type of grammatical action at a distance reveals the highly abstract nature of syntax. Two phrases that have very different surface forms, and systematically appear in different places in a sentence, nonetheless each satisfy the verb–object dependency. And these dependencies can hold across arbitrarily large distances and over certain internally complex structures (*iii*), two properties that reveal the high degree of computational power of the human linguistic system (5–11).

Here, we investigate some of the first stages in the development of this system. Whereas hierarchical and recursive structure has been the focus of much prior work in this area (12–17), we turn our attention to abstract syntactic dependencies—a central domain of syntax that has been less explored in early development. Specifically, we ask whether infants share our ability to represent grammatical action at a distance, even before they regularly combine words into sentences in their own speech. This helps illuminate whether some of the core computational properties that characterize syntax are present early in cognitive development, setting the stage for further questions regarding the origins of these computational capacities.

Answering this question requires a method for identifying syntactic representations in infants who are not yet producing many sentences of their own. We often test syntax by asking how children understand sentences (18–21), but this runs the risk that an infant’s apparent comprehension of a sentence in context may depend on immature syntactic representations that mimic the correct interpretations. Another approach has examined when infants detect dependencies in sentences they hear without relying on interpretation (22–28). However, in these cases, surface and syntactic dependencies are often confounded. A key to isolating syntactic dependencies independent of meaning is to show that infants are aware of complementary distribution patterns like those in *i* to *iv*. For instance, we need to test when infants know that a local object and an object *wh*-phrase cannot co-occur because they both enter into the same abstract dependency with the verb. We provide this test here.

## Nonlocal Dependencies in Infancy

Languages vary in how syntactic dependencies are expressed, so children must learn to recognize them in the particular language they are exposed to. Previous work has found that infants in their second year of life have the ability to recognize dependencies between nonadjacent sounds or morphemes in both natural and artificial languages (22–28). For instance, 18-mo-old English learners show awareness of the dependency between *is* and -*ing* in sentences like *The archeologist is digging for treasures* (26, 27). However, it is difficult to determine how infants represent these dependencies. In these studies, infants might be tracking the co-occurrence of particular sounds, like “is” and “ing,” or they might be representing a more abstract underlying relation, like the one between auxiliary *be* and a verb in the progressive aspect (26, 27) (see also refs. 24 and 25).

We turn to a different case study that allows for better separation of these possibilities: the nonlocal predicate-argument dependencies found in *wh*-questions. In English, *wh*-phrases (e.g., *what*) have different surface forms than subjects and objects that are local to the verb (e.g., *the pizza*). They also have different distributions. For instance, *wh*-phrases overwhelmingly occur clause initially, whereas local objects canonically occur after the verb. So, for infants to recognize that the same verb–object dependency is present locally in a simple transitive sentence (*i*) and at a distance in an object *wh*-question (*iii*), they need to abstract away from these distinct surface properties. It is not enough to represent co-occurrences of specific sounds or even to know that a clause-initial word like *what* will eventually be followed by a verb. Instead, infants must represent the nonlocal dependency abstractly, as an instance of the same relation established locally between a verb and its direct object.

To date, data on infants’ *wh*-question representations comes from two primary sources. The first is evidence about the utterances that infants produce. The second is evidence about what interpretations they assign to utterances of sentences they hear. Both of these measures speak only indirectly to the question of how infants represent the structures of the sentences they produce or understand. Argument *wh*-questions begin to appear in infants’ telegraphic speech around the age of 20 mo (29, 30), but as comprehension generally precedes production (19), this evidence does not tell us when infants first represent these questions in an adult-like way. On the comprehension end, infants around 15 mo have sometimes been shown to respond appropriately to a *wh*-question (18, 20, 21). However, such responses may depend on identifying local subjects and objects in concert with pragmatic reasoning, rather than representing nonlocal syntactic dependencies themselves.

For example, previous comprehension studies used preferential looking tasks with infants from 13 to 20 mo in which infants saw an event or a series of events (e.g., a brown dog bumps into a cat, who then bumps into a black dog) and were then asked a question about those events (e.g., *Which dog did the cat bump?*). Two possible answers (e.g., the two dogs) were shown on the screen; if infants looked more at the image corresponding to the correct answer, one might conclude that infants understood the question and hence represented it in a syntactically adult-like fashion. However, refs. 7 and 20 argued that success on such tasks does not depend on correct syntactic representation of the dependencies in these questions. If infants recognized that a question was being asked and understood that *the cat* was the subject and *bump* was the verb, they might be inclined to look at the dog that the cat bumped, even without representing the *wh*-phrase *which dog* as the object of the verb. In order to determine whether infants represent the nonlocal predicate-argument dependencies in these sentences, then, it is necessary to show that they are aware of the complementarity between the *wh*-phrase and the local object of the verb as illustrated in *iii* and *iv*.

**Fig. 1.**
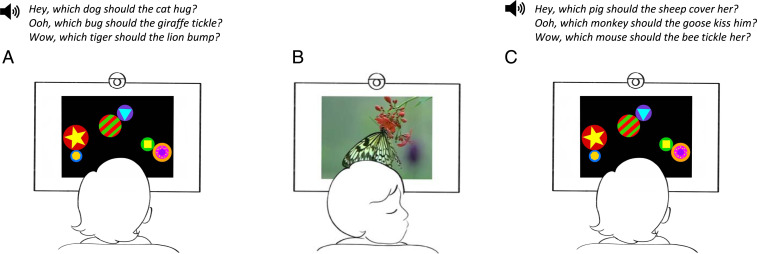
Schematic depiction of test trials in Experiments 1 to 4 illustrating the *wh*-question condition. (*A*) An infant watches an abstract video of rotating shapes on a widescreen television. While the video plays, the infant hears up to six *wh*-questions; infants assigned to the declarative condition hear up to six declarative sentences. An experimenter in a separate room live-codes infants’ eye fixations by monitoring a video feed from a camera located above the television. When all six test sentences are played or the infant looks away from the screen for more than two seconds (*B*), the trial ends and an attention-getter stimulus is displayed. When the infant reorients toward the screen (*C*), the next trial begins. Infants hear alternating trials of sentences without objects (*A*) and with objects (*C*).

The current research introduces a test that probes this complementarity directly (Fig. 1). Using infants’ listening time in the absence of a referential context as a probe, we ask whether infants’ behavior differentiates *wh*-questions with no local object from those that do contain a local object, essentially providing a comparative measure of acceptability. In our task, infants listened to blocks of auditorily presented sentences, either *wh*-questions (*v*) or declaratives (*vi*). Infants in both conditions heard sentences with and without direct objects after the verb, presented in alternating trials. All sentences contained transitive verbs that were among the most likely to be known by children at 16 mo of age (31). While they listened, we presented a video of abstract shapes moving on the screen, and we recorded looking time toward the screen as a measure of infants’ interest in these sentences (32, 33). If infants looked away for more than 2 s, the current trial ended. When they reoriented toward the screen, a new trial was initiated.

v. a. Which dog should the cat hug?
b. *Which dog should the cat hug him?
vi. a. *A dog! The cat should hug.
b. A dog! The cat should hug him.

In this design, the acceptability of a local object is flipped across the two critical pairs. The verb *hug* needs a direct object, and *which dog* can serve as this object nonlocally in a *wh*-question. This makes the local object *him* unacceptable in the *wh*-questions in *v*: there are too many objects. However, the local object is required in the declaratives in *vi*. If infants know these facts, then their preferences for the (a) versus the (b) sentences should likewise be flipped across these sentence pairs: their relative preference for sentences with local objects should be opposite in *wh*-questions and declaratives. On the other hand, suppose infants only know that the verbs in our study require objects but do not represent the *wh*-phrases as satisfying this requirement nonlocally. Under this hypothesis, their patterns of preference should be the same across these sentence pairs. That is, if they prefer sentences with local objects in declaratives, then they should likewise prefer them in *wh*-questions.^†^

## Results

In our first experiment, we tested 18-mo-old English-learning infants. This age was chosen for two reasons. First, given the general tendency for comprehension to precede production in language acquisition, we suspected that infants might represent *wh*-questions as such in the months leading up to their first production of these questions, around 20 mo of age (29, 30). Second, the listening preference procedure has been used successfully with infants in a wide age range, from 8 to 30 mo, and is well-suited to the behavioral capacities of infants at 18 mo and younger (28, 32, 38). One group of infants (*n* = 16) heard sentences like *v*, and the other group (*n* = 16) heard sentences like *vi*.

**Fig. 2.**
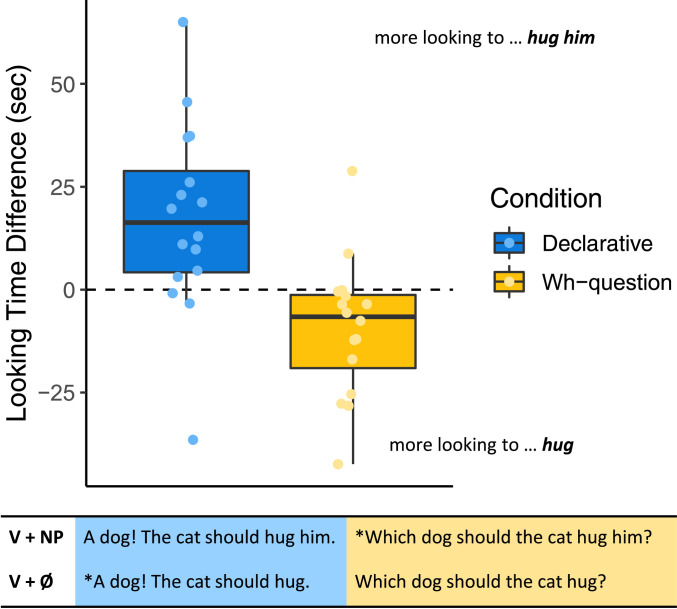
Looking-time preferences of 18 mo olds in Experiment 1 who heard declarative sentences (*n* = 16) shown in blue and *wh*-questions (*n* = 16) in orange. Dots show individual participant preferences, plotted as a difference in total looking time in seconds across the experiment for trials with local objects, minus total looking time for trials without local objects. Positive numbers indicate a preference for trials with local objects, and negative numbers indicate a preference for trials without local objects. Zero, shown by the dashed line, indicates no preference. The solid black lines in each boxplot indicate median preference scores, with whiskers extending to preference scores at most 1.5 times the inter-quartile range above the third quartile or below the first quartile.

We plot infants’ listening time preferences in Fig. 2 as differences in seconds between total time spent listening to sentences with local objects and total time spent listening to sentences without. Infants’ raw total listening times to each sentence type are plotted in *SI Appendix*, Fig. S1.

We analyzed these data by first comparing infants’ preferences across sentence types (declarative versus *wh*-question). Consistent with our predictions, we found that infants’ preferences for *wh*-questions (*M* = −9.35, *SE* = 4.29) and declaratives (*M* = 17.23, SE = 5.82) were significantly different from each other (Welch’s *t*(27.26) = 3.70, *P* < 0.001, *d* = 1.31). We then asked whether infants showed preferences that were significantly different from zero for each sentence type. We found that infants’ preferences were reliable in both conditions. Infants who heard declaratives showed a significant preference for sentences with local objects (*t*(15) = 2.96, *P* < 0.01, *d* = 0.74). Infants who heard *wh*-questions showed a significant preference against sentences with local objects (*t*(15) = −2.23, *P* < 0.04, *d* = −0.56), instead preferring sentences without local objects. That is, in both conditions, infants preferred to listen to grammatical sentences.^‡^

This pattern of results shows that 18 mo olds are aware of the complementarity between the *wh*-phrase and the local object of the verb, consistent with the hypothesis that infants at this age represent the abstract dependency between a *wh*-phrase and a verb located several words downstream. Because they prefer sentences in which the verb occurs without its direct object only when a *wh*-phrase occurs earlier in the sentence, we can infer that infants treat the *wh*-phrase as satisfying the verb’s requirement for a direct object.

This result puts us in position to ask whether the same pattern holds in younger children. Prior literature proposed that although infants as young as 15 mo behave as if they understand *wh*-questions, they do not represent the grammatical dependency between the *wh*-phrase and the verb (18, 20). In our second, third, and fourth experiments, we investigated this proposal by testing groups of 14 mo olds (Experiment 2), 15 mo olds (Experiment 3), and 17 mo olds (Experiment 4). The procedures were identical to that used in Experiment 1.

**Fig. 3.**
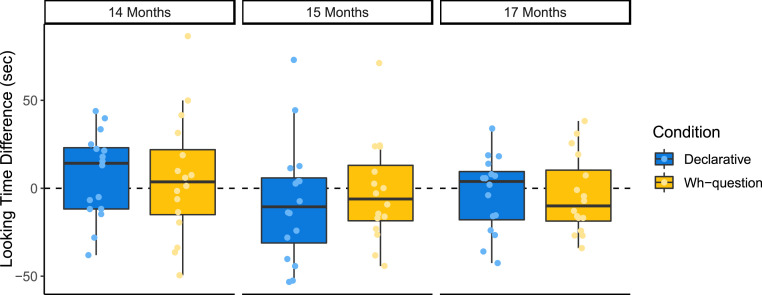
Total looking times for trials with local objects minus trials without local objects for 14 mo olds (Experiment 2), 15 mo olds (Experiment 3), and 17 mo olds (Experiment 4). For each experiment, preferences of infants who heard declarative sentences (*n* = 16) are shown in blue, and infants who heard *wh*-questions (*n* = 16) are shown in orange.

Listening preferences for Experiments 2 to 4 are displayed in Fig. 3, and raw listening times to each sentence type are plotted in *SI Appendix*, Fig. S2. Our analyses found no significant preferences for declaratives or *wh*-questions in each experiment and no differences in listening preferences across sentence types. Specifically, infants did not show significantly different preferences for declaratives and *wh*-questions at 14 mo (*M*_*D*_ = 7.26, *SE*_*D*_ = 6.09, *M*_*WH*_ = 5.78, *SE*_*WH*_ = 8.71, Welch’s *t*(27.69) = 0.72, *P* < 0.48, *d* = 0.05), 15 mo (*M*_*D*_ = −7.84, *SE*_*D*_ = 8.57, *M*_*WH*_ = −2.26, *SE*_*WH*_ = 7.16, Welch’s *t*(29.08) = −0.50, *P* < 0.62, *d* = −0.18), or 17 mo (*M*_*D*_ = −3.18, *SE*_*D*_ = 5.38, *M*_*WH*_ = −4.10, *SE*_*WH*_ = 5.58, Welch’s *t*(29.96) = 0.12, *P* < 0.91, *d* = 0.04). For each experiment, we then compared infants’ preference scores to zero in both conditions. Infants who heard declaratives did not show a significant preference for or against local objects at 14 mo (*t*(15) = 1.19, *P* < 0.25, *d* = 0.30), 15 mo (*t*(15) = −0.92, *P* < 0.37, *d* = −0.23), or 17 mo (*t*(15) = −0.59, *P* < 0.56, *d* = −0.15). Similarly, we found no significant preference for or against local objects in *wh*-questions at 14 mo (*t*(15) = 0.66, *P* < 0.52, *d* = 0.17), 15 mo (*t*(15) = −0.32, *P* < 0.76, *d* = −0.08), or 17 mo (*t*(15) = −0.73, *P* < 0.47, *d* = −0.18).

Thus, the 14 to 17 mo olds in Experiments 2 to 4 do not show the same pattern of behavior as the 18 mo olds in Experiment 1. To ask whether infants’ listening preferences significantly differ across this age range, we combined the data across all four experiments. We analyzed infants’ listening times during each trial using a linear mixed-effects model with fixed effects of age in days, condition (declarative versus *wh*-question), local object, and experiment block (details in *Materials and Methods*). For this and the following analyses, coefficients and full regression results can be found in *SI Appendix*, Tables S1–S3. Consistent with our predictions, our linear mixed-effects regression revealed a significant interaction of age, condition, and local object (*t*(1,140) = −1.98, *P* < 0.05), with no other significant effects. To visualize this interaction, we plot model predictions in *SI Appendix*, Fig. S3.

In order to determine whether this interaction is present across the entire age range, we next conducted linear mixed-effects model analyses for the younger infants (14 to 15 mo olds) and the older infants (17 to 18 mo olds) in separate groups. For the younger group, no significant main effects or interactions were found. For the older group, we found a significant interaction of condition and local object (*t*(564) = 2.03, *P* < 0.05) and a significant interaction of age, condition, and local object (*t*(564) = −2.10, *P* < 0.04), with no other significant effects. Model predictions for both age groups are plotted in *SI Appendix*, Fig. S4. These findings indicate that infants’ different listening patterns for *wh*-questions and declaratives emerge in the upper end of our age range, developing between 17 and 18 mo.

These results are consistent with the hypothesis that 14 and 15 mo olds are unaware that a *wh*-phrase can function as the object of a verb from a distance and that this awareness emerges between 17 to 18 mo of age. In the aggregate, only the 18 mo olds we tested differentiated sentences that were missing objects because of an earlier *wh*-phrase from those that were simply missing objects. We therefore do not find evidence that younger infants represent the grammatical dependency between the *wh*-phrase and the verb.^§^

## Discussion

A central property of syntax is its ability to encode abstract grammatical dependencies that can hold at a distance. When does this property emerge in cognitive development? This work contributes two findings to address this question. First, we show that infants have the capacity to represent a nonlocal syntactic dependency at 18 mo, before they regularly produce full sentences of their own. We find that 18 mo olds represent the nonlocal predicate-argument dependency inherent in a *wh*-question abstractly, as equivalent to the grammatical relation between a verb and its local direct object. This suggests that these abstract representations underlie infants’ first productions of *wh*-questions as well as their success in comprehension tasks after this age (18, 21, 29, 30). Because prior work examining *wh*-questions depended on interpretation, it spoke only indirectly to infants’ syntactic representations of these sentences. Here, by removing the question of interpretation, we get a clearer signal of infants’ syntactic knowledge.

Second, we show that younger infants do not represent the dependencies in these sentences in the same way. Infants as young as 15 mo may show an ability to appropriately respond to *wh*-questions (18, 20, 21), but we do not find evidence that they recognize the complementarity between a *wh*-phrase and a local object of the verb. While further work is needed to explore this null result, a plausible explanation is development in syntactic knowledge: infants younger than 18 mo may not represent these questions as having a nonlocal grammatical dependency. This pattern is thus consistent with the hypothesis from prior work that a parse of only local argument relations can lead to the appearance of the understanding of *wh*-questions.

However, further work is needed to isolate syntactic knowledge from other factors that may have contributed to younger infants’ behavior. For instance, infants were familiarized with the nouns and verbs in our study before hearing our test sentences (see *Materials and Methods*), and it is possible that this familiarization phase may have affected infants’ processing of the test sentences in variable ways at different ages.^¶^ It is also possible that the *which-*phrases in our *wh*-questions, included for direct comparison to the sentences tested in prior preferential looking studies (18, 20), may have introduced syntactic or semantic complexity that younger infants found difficult to process. Future work with different materials, including simple *wh*-phrases like *who* or *what*, aims to differentiate these possibilities.

What learning mechanisms could be responsible for the change that we see between 14 and 18 mo? One possibility, consistent with a broader literature on the role of expectation violation in cognitive development (39–42), is that acquisition of local argument relations may be a necessary precursor to the identification of nonlocal dependencies in the target language. In a language with stable word order, a learner’s ability to recognize arguments in their canonical positions and recognize when they are unexpectedly missing in those positions could compel the learner to look elsewhere in the sentence to find the missing argument. Identifying the particular surface forms that co-occur with missing arguments of verbs could allow a learner to recognize that *what* and *which*, for example, head phrases that realize the missing argument nonlocally (18, 20, 30, 43). Ref. 43 shows computationally that this mechanism is feasible, but further work is required to determine whether it is the mechanism that learners use in acquiring a language like English. An important direction for future work, therefore, is studying more closely the development of argument structure knowledge and its interaction with *wh*-question acquisition before 18 mo. While some work suggests knowledge of argument structure emerging early in the second year of life (44, 45), specifying how that knowledge interacts with the acquisition of *wh*-questions remains an important goal.

Our case study contributes to the broader literature on the development of syntax as a particular kind of computational system: one that is able to compute potentially unbounded dependencies over abstract structural configurations and to recursively nest these dependencies (1, 2, 5–11). Much of this literature has focused on the second of these properties, recursion, as the characteristic signature of this system (12–17). The current work shows how the first property, the dependencies themselves, provides a promising avenue for studying syntax acquisition. The finding that young infants are capable of representing a nonlocal dependency as structurally equivalent to a local dependency tells us that a core computational property of syntax emerges very early in development, when infants’ speech is still highly telegraphic.

An important direction for future work is probing the finer details of how 18 mo olds represent this dependency: whether as potentially unbounded and defined over particular structural configurations but not others (9, 10). In particular, the current finding does not speak to the question of whether infants’ representations of this dependency involve hierarchical structure. While our results show that infants recognize that transitive verbs can occur without their objects only when a *wh*-phrase occurs earlier in the sentence, further work is needed to demonstrate that they represent the *wh*-phrase and the verb in the specific structural configurations in which they would appear in the adult grammar. One way to address this question is to ask whether infants incrementally parse and interpret the *wh*-phrase in direct object position during online sentence processing.

Finally, the finding that abstract syntactic representations emerge during the second year of life suggests an important role for learning from experience and makes contact with the debate over how learning interacts with a learner’s resources for representing linguistic input. On one view, the representational capacity for computing abstract syntactic dependencies gradually develops in childhood; infants’ earliest sentence representations lack these dependencies because their minds gain the capacity to represent them through the process of learning from their input (29, 46–49). On an alternative view, human cognition includes this core representational capacity for abstract syntactic dependencies, but its expression in behavior depends on a mechanism for identifying how such dependencies are realized in the local linguistic environment (1, 50–54). While the current work does not differentiate these alternatives, the findings in this paper place an important benchmark in the timeline of syntactic development, thereby constraining any theory of when and how syntactic knowledge is acquired.

## Materials and Methods

All infant experiments were approved by the University of Maryland Institutional Review Board. Informed written consent was obtained by a participant’s caregiver before an experiment began. The norming experiment with adult participants was certified by the Institutional Review Board of the University of California, Los Angeles. Participants gave informed consent before viewing any study materials.

### Participants.

Participants were recruited from the local area with the criterion that they heard English during at least 80% of their waking hours. Participants in Experiment 1 included 32 infants (15 male) between the ages of 18;0 and 18;31 (mean = 18;12). An additional nine infants were tested but not included in the sample due to failing to complete the full familiarization phase as described under *Procedure* (two), failing to complete the full test phase (five), fussiness or inattentiveness (one), or parental interference during the study (one). Participants in Experiment 2 included 32 infants (19 male) between the ages of 14;0 and 14;29 (mean = 14;13). An additional 18 infants were tested but not included in the sample due to failing to complete the full familiarization phase (three), failing to complete the full test phase (seven), or fussiness or inattentiveness (eight). Participants in Experiment 3 included 32 infants (16 male) between the ages of 15;0 and 15;28 (mean = 15;14). An additional 10 infants were tested but not included in the sample due to failing to complete the full test phase (three) or fussiness or inattentiveness (seven). Participants in Experiment 4 included 32 infants (17 male) between the ages of 17;1 and 17;28 (mean = 17;14). An additional 18 infants were tested but not included in the sample due to failing to complete the full familiarization phase (four), failing to complete the full test phase (five), fussiness or inattentiveness (seven), or parental interference during the study (two). The exclusion criteria for fussiness or inattentiveness were conservative and applied at time of testing. The experimenter made a note if an infant cried or moved to such an extent that eye gaze could not be reliably coded, and data from that infant were then excluded from analysis.

### Materials.

Infants heard 25-s trials presenting six sentences, one for each of the six verbs tested (*kiss*, *hug*, *tickle*, *bump*, *hit*, and *cover*). These verbs were chosen to be highly transitive, familiar to infants at 16 mo of age (31), and as similar as possible to the verbs tested in previous preferential looking experiments (18, 20). Verbs were presented in a pseudorandom order with different animals as NP arguments. These nouns were the 24 animals reported in ref. 31 with the highest rates of production and comprehension at 16 mo. According to ref. 31, 9% of American 16 mo olds produce and (approximately) 70% comprehend^#^ the tested verbs, on average; 21% of 16 mo olds produce, and 55% comprehend the tested nouns, on average.

*Wh-*questions used *which* as the *wh*-word in order to allow as close a comparison as possible to previous experiments (18, 20). The number and order of lexical NPs was matched in the *wh*-question and declarative conditions by using pronominal objects (*him* and *her*) instead of lexical NP objects. NP fragments (e.g., *A tiger!*) preceded the declarative sentences and introduced a referent for these pronouns. A prosodic break indicated an utterance boundary between each fragment and the following sentence.

All sentences were recorded by a female native speaker of American English using child-directed speech. In order to maintain natural-sounding prosody, all ungrammatical conditions were created by splicing together two grammatical sentences. Ungrammatical *wh*-questions were created by splicing a grammatical *wh*-question with a causative sentence (*Which tiger should the lion hug + I made the lion hug him = Which tiger should the lion hug him*). Ungrammatical declaratives were created by splicing a grammatical declarative with an embedded question (*The lion should hug him + I know who the lion should hug = The lion should hug*). The modal *should* was used in order to avoid differences in verbal morphology across sentence types. The full set of stimuli sentences is listed in *SI Appendix*, Tables S4–S6.

Audio stimuli were edited using Adobe Audition and Praat and concatenated with variable 750 to 1,000 ms of silence between sentences. To ensure that the splices in the ungrammatical sentences were not detectable, we collected judgments from a sample of 30 adult participants over Amazon Mechanical Turk. Participants listened to short audio clips from both our grammatical and ungrammatical sentences and were asked to indicate which clips they thought were edited (details in *SI Appendix*, *SI Materials and Methods*). Participants were at chance at discriminating edited from unedited clips (mean *d*' = −0.07, *SE* = 0.05; comparison to zero: *t*(29) = −1.28, *P* < 0.21).

Audio for each trial was combined with one of two videos of animated, slowly rotating shapes in Adobe Premiere. One of these videos was used during the familiarization phase of the experiment and one during the test phase. A silent video of a butterfly on a leaf was separately edited for use as an attention-getter stimulus.

### Procedure.

During an experiment, infants sat on a parent’s lap or a highchair positioned 6 ft away from a 51” widescreen television. Parents listened to music played over noise-cancelling headphones and were instructed not to talk to their children or direct their attention. Stimuli were played using the Habit program (55). A camera located above the television was used to video-record the experiment. The camera feed was connected to a video monitor in a separate room to allow an experimenter in a separate room to live-code infants’ eye fixations. The experimenter was not able to hear the audio for the experiment and therefore was blind to the particular trial type that the infant was hearing.

Each experiment began by displaying the attention-getter stimulus. Once the infant fixated on the attention getter, the experimenter initiated the first trial. During each trial, the experimenter pressed a key on the computer to record when the infant was looking at the screen and released the key as soon as the infant looked away. A trial ended after the full 25-s duration of the audio stimulus or after the computer program registered that the infant looked away from the screen for more than 2 s continuously. At the end of a trial, the attention-getter stimulus was displayed. The next trial was initiated as soon as the infant reoriented back toward the screen.

The experiment had two phases. During the familiarization phase, infants were familiarized to at least 72 s of the six test verbs in basic transitive clauses with direct objects (*SI Appendix*, Table S1). Because young infants’ prior experience with the experimental verbs may vary, this phase was intended to aid lexical processing during our task by facilitating retrieval of these lexical items and their argument structure from memory. Familiarization sentences had the same structure as the local object sentences presented in the declarative test condition but did not include any of the same sentences presented at test. In order to ensure that infants in the declarative condition were exposed to sufficiently novel stimuli at test, different videos of rotating shapes were used for the familiarization and test phases of the experiment for both conditions.

Four 25-s familiarization trials were prepared, each containing six sentences with the test verbs presented in a pseudorandom order. The order of familiarization trials was randomized across participants. The familiarization phase ended after the trial during which an infant reached the 72-s threshold of looking time. If this threshold was not reached during the first presentation of the familiarization trials, they repeated in a random order for up to 12 trials total. Infants who did not reach 72 s of looking time over the course of 12 familiarization trials were excluded from the final sample.

After the familiarization phase, the experiment proceeded to the test phase. At test, infants heard 12 trials of declarative sentences or *wh*-object questions (a between-subjects factor), alternating between trials with local objects and trials without (a within-subjects factor) (*SI Appendix*, Tables S2–S3). Infants in each condition were randomly assigned to one of four lists, counterbalancing two factors across participants. The grammaticality of the first test trial was counterbalanced by presenting half of participants with a grammatical first test trial and half with an ungrammatical test trial in their condition, and test trial order was counterbalanced by reversing the order of trial presentation for half of the participants. Infants who did not complete all 12 test trials or became excessively fussy over the course of the experiment were excluded from the final sample.

### Linear Mixed-Effects Models.

For analyses across multiple age groups, we constructed linear mixed-effects models using the *lme4* package in R (56). Our dependent measure was total looking time in seconds for each trial. Fixed effects included age in days, condition (declarative versus *wh*-question), trial type (local object versus no local object), and experiment block (whether a trial fell within the first, second, or third block of four trials within the test phase) as well as all interaction terms. Factor contrasts were sum coded. In order to account for individual subject differences in listening time over the course of the experiment, our full model included a random intercept for subject and a random slope for block. Because this model fit was singular for our analysis of the 14- to 15-mo-old age group, that model included only the random intercept for subject. Significance tests were conducted using Satterthwaite’s approximation for degrees of freedom, implemented using *lmerTest* (57), which has been shown to minimize type I error (58).

## Supplementary Material

Supplementary File

## Data Availability

Anonymized data have been deposited in the Open Science Framework database (https://osf.io/4sz9w/) (59). All other study data are reported in the main text and *SI Appendix*.

## Footnotes

*Denotes unacceptability.

^†^It is complicated to predict the absolute direction of listening- or looking-time preferences in a given task using this method, and preference directions are known to vary by age (34–37). For this reason, what matters in our design is not the absolute direction of infants’ preferences for sentences with or without local objects but whether their relative preferences are the same or different in declaratives versus *wh*-questions at any particular age.

^‡^The main findings from these *t* tests, and the *t* tests reported for Experiments 2 to 4, are the same if conducted on preference scores calculated from log-transformed looking times.

^§^Although 17 mo olds in the aggregate did not show evidence of having acquired these representations, the significant two-way interaction of condition and local object found for 17 to 18 mo olds leaves open the possibility that this acquisition takes place later than 17 mo but earlier than 18 mo. We leave this possibility for future work to investigate.

^¶^In particular, a reasonable concern is that the similarity between our familiarization sentences and the declarative test sentences may have introduced differences in task difficulty for infants in the declarative versus *wh*-question conditions. However, infants’ overall raw listening times were similar for both sentence types at test: our linear mixed-effects model analyses found no main effect of condition nor interaction of condition and age across the 14- to 18-mo-old age range nor within the 14- and 15-mo-old or 17- and 18-mo-old age groups analyzed separately (*SI Appendix*, Tables S1–S3). This suggests that task demands did not differ substantially across conditions.

^#^Ref. 31 does not report comprehension data for the verb *cover*, so this average comprehension rate includes data only for *kiss*, *hug*, *tickle*, *bump*, and *hit*.
