# Supplementary material for: Eighteen-month-old infants represent nonlocal syntactic dependencies
Source: Proc Natl Acad Sci U S A. 2021 Oct 4;118(41):e2026469118. doi: 10.1073/pnas.2026469118 (PMC8521675; doi:10.1073/pnas.2026469118)
Supplement: Supplementary File [file pnas.2026469118.sapp.pdf]

PNAS
www.pnas.org

Supplementary Information for
18-Month-Old Infants Represent Non-Local Syntactic Dependencies

Laurel Perkins, Jeffrey Lidz

Corresponding author: Laurel Perkins
Email: perkinsl@ucla.edu

This PDF file includes:

Supplementary text
Figures S1 to S4
Tables S1 to S6
SI References

Supplementary Information Text

SI Materials and Methods

In order to norm our audio materials, we recruited 40 adult participants over Amazon Mechanical Turk. Each participant was paid $2.50 for completing the study. Participants were required to have completed 100 previous tasks on the platform with a lifetime approval rating of 95% or greater, and to have an IP address in the United States. Participants clicked on a link that directed them to an anonymous online survey created using PCIbex (1). The survey took approximately 10-15 minutes to complete.

Participants were presented with short audio clips from both the grammatical and ungrammatical sentences in Experiments 1-4. Clips were prepared by selecting a region containing the splices in the ungrammatical sentences and the corresponding region in the grammatical sentences. These regions contained the modal *should*, the subject, and the verb (e.g. declarative: *the lion should hug*; *wh*-question: *should the lion hug*). Identical words were present in the spliced and unspliced variant of each clip. Clips were edited in Praat with a 0.1 sec fade applied to the beginning and end.

Participants were randomly assigned to one of two stimulus lists, each of which contained 36 spliced and 36 unspliced clips, with order of presentation randomized for each participant. No participant heard the same clip in both its spliced and unspliced variant. Participants were told that some of these clips were from recordings of a person's natural speech, and other clips were created by splicing together recordings of two different sentences. They were instructed to listen for splices in the middle of these clips, and to click a radio button for each clip indicating whether they thought there was a splice in the middle, or whether it was naturally-produced. Participants were able to replay each clip as many times as they liked.

One-third of trials were followed by a probe question asking participants to recall words in the clips (e.g. *Did you hear 'frog'?*). The correct answer was 'yes' for half of these questions. Participants who did not answer at least 80% of these probe questions correctly were excluded from the final sample; this resulted in the exclusion of 10 participants' data. Of the remaining 30 participants, mean accuracy on these probes was 96%. Trials on which these participants answered the following probe question incorrectly were excluded from analysis; this resulted in the removal of 28 total trials from 12 participants.

Data were analyzed by calculating each participant's percent accuracy and d' on discriminating spliced from unspliced clips. Mean accuracy was 49% (*SE*: 0.9%), which was not significantly different from chance (50%) ($t(29) = -1.14$, $p < 0.26$). Mean d' was -0.07 (*SE:* 0.05), which was not significantly different from zero ($t(29) = -1.28$, $p < 0.21$). This indicates that each participant's hit rate (rate of guessing "spliced" for clips that were actually spliced) was not significantly different from the rate of false alarms (rate of guessing "spliced" for unspliced clips).

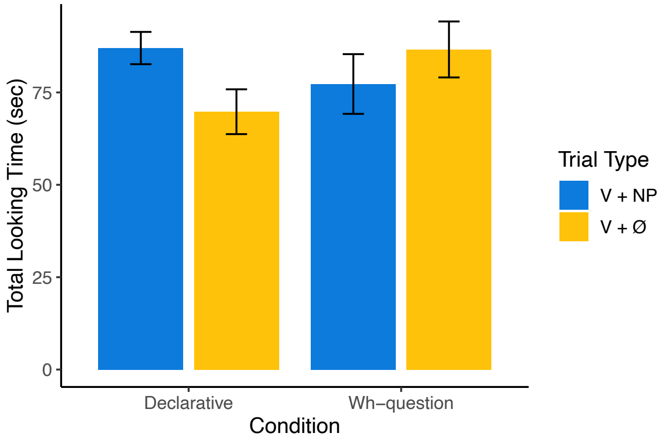

Figure S1. Mean total looking time at test for 18-month-olds in Exp. 1 who heard declarative sentences (n = 16), shown on the left, and *wh*-questions (n = 16), shown on the right. Blue bars indicate trials with local objects; orange bars indicate trials without local objects. Error bars represent ±1 SEM.

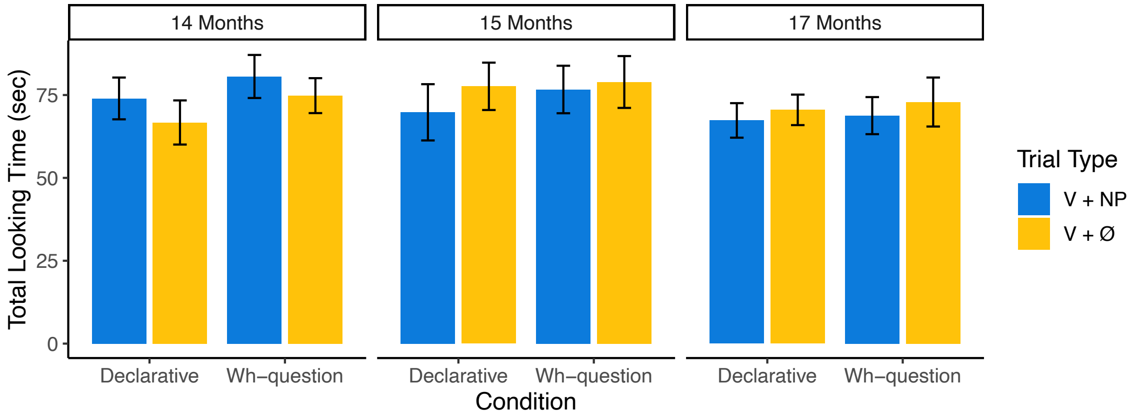

Figure S2. Mean total looking time at test for 14-month-olds (Exp. 2), 15-month-olds (Exp. 3), and 17-month-olds (Exp. 4). For each experiment, preferences of infants who heard declarative sentences (*n* = 16) are shown on the left, and infants who heard *wh*-questions (*n* = 16) are shown on the right. Blue bars indicate trials with local objects; orange bars indicate trials without local objects. Error bars represent ±1 SEM.

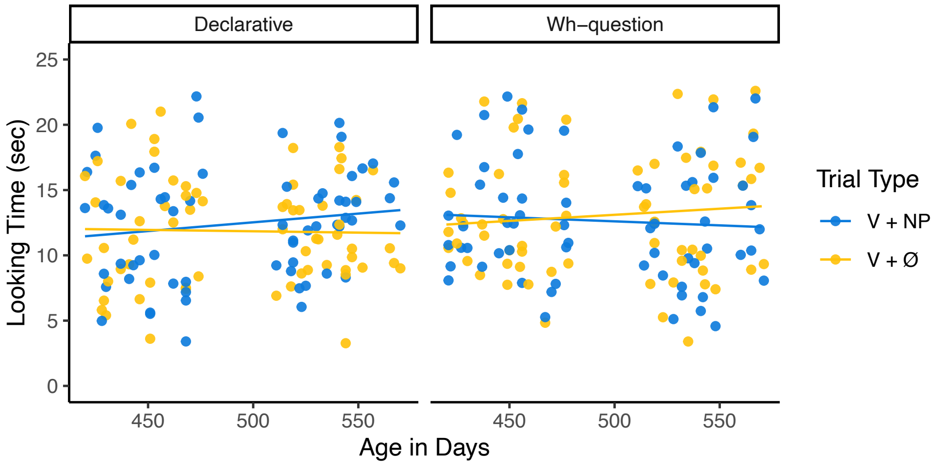

Figure S3. Mean looking time per test trial for all infants in Exp. 1-4 who heard declarative sentences (n = 64), shown on the left, and *wh*-questions (n = 64), shown on the right. Blue dots indicate trials with local objects; orange dots indicate trials without local objects. Lines show predictions of linear mixed effects model regressions.

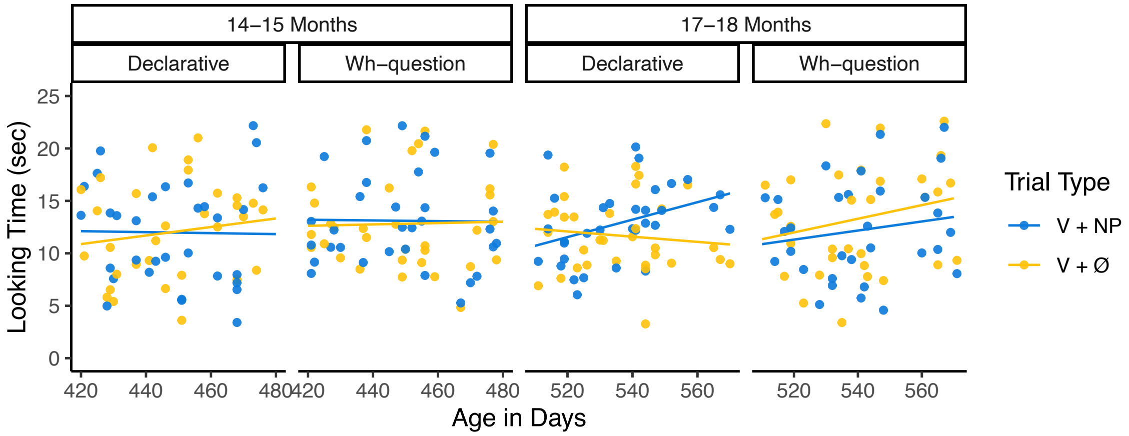

Figure S4. Mean looking time per test trial for 14- to 15-month-olds (Exps. 2-3) and 17- to 18-month-olds (Exps. 1 and 4). Within each age group, looking times for infants who heard declarative sentences (*n* = 32) are shown on the left, and infants who heard *wh*-questions (*n* = 32) are shown on the right. Blue dots indicate trials with local objects; orange dots indicate trials without local objects. Lines show predictions of linear mixed effects model regressions conducted over each age group separately.

Table S1. Effects Summary: Linear Mixed Effects Model Regression, 14- to 18-Month-Olds

Fixed effect	*Estimate*	*Std. Error*	*Df*	*t*	*p*
(Intercept)	10.72	3.49	124	3.07	**0.003**
Condition	-1.35	3.49	124	-0.39	0.698
Age	0.00	0.01	124	0.51	0.609
Object	-0.06	1.94	1140	-0.03	0.977
Block1	-1.50	2.94	124	-0.51	0.610
Block2	3.28	3.08	124	1.06	0.288
Condition:Age	0.00	0.07	124	0.29	0.772
Condition:Object	3.58	1.94	1140	1.85	0.065
Age:Object	0.00	0.04	1140	0.01	0.996
Condition:Block1	-0.20	2.94	124	-0.07	0.945
Condition:Block2	0.80	3.08	124	0.26	0.794
Age:Block1	0.01	0.06	124	1.58	0.116
Age:Block2	-0.01	0.06	124	-0.96	0.337
Object:Block1	-0.91	2.74	1140	-0.33	0.739
Object:Block2	-1.15	2.74	1140	-0.42	0.675
Condition:Age:Object	-0.01	0.04	1140	-1.98	**0.048**
Condition:Age:Block1	0.00	0.06	124	0.17	0.863
Condition:Age:Block2	0.00	0.06	124	-0.22	0.827
Condition:Object:Block1	-1.48	2.74	1140	-0.54	0.588
Condition:Object:Block2	0.03	2.74	1140	0.01	0.992
Age:Object:Block1	0.00	0.06	1140	0.31	0.759
Age:Object:Block2	0.00	0.06	1140	0.67	0.503
Condition:Age:Object:Block1	0.00	0.06	1140	0.56	0.579
Condition:Age:Object:Block2	0.00	0.06	1140	0.05	0.961

Table S2. Effects Summary: Linear Mixed Effects Model Regression, 14- to 15-Month-Olds

Fixed effect	*Estimate*	*Std. Error*	*Df*	*t*	*p*
(Intercept)	8.09	12.22	60	0.66	0.508
Condition	-4.16	12.22	60	-0.34	0.734
Age	0.01	0.03	60	0.36	0.719
Object	-6.24	6.83	684	-0.91	0.361
Block1	15.37	9.67	684	1.59	0.112
Block2	-7.89	9.67	684	-0.82	0.415
Condition:Age	0.01	0.03	60	0.30	0.763
Condition:Object	-3.90	6.83	684	-0.57	0.568
Age:Object	0.01	0.02	684	0.91	0.365
Condition:Block1	13.06	9.67	684	1.35	0.176
Condition:Block2	-1.75	9.67	648	-0.18	0.856
Age:Block1	-0.03	0.02	684	-1.34	0.182
Age:Block2	0.02	0.02	684	0.89	0.379
Object:Block1	-2.57	9.67	684	-0.27	0.790
Object:Block2	-9.44	9.67	684	-0.98	0.329
Condition:Age:Object	0.01	0.02	684	0.59	0.559
Condition:Age:Block1	-0.03	0.02	684	-1.34	0.182
Condition:Age:Block2	0.00	0.02	684	0.21	0.837
Condition:Object:Block1	-3.59	9.67	684	-0.37	0.710
Condition:Object:Block2	-14.90	9.67	684	-1.54	0.123
Age:Object:Block1	0.01	0.02	684	0.26	0.793
Age:Object:Block2	0.02	0.02	684	1.04	0.301
Condition:Age:Object:Block1	0.01	0.02	684	0.36	0.720
Condition:Age:Object:Block2	0.03	0.02	684	1.58	0.114

Table S3. Effects Summary: Linear Mixed Effects Model Regression, 17- to 18-Month-Olds

Fixed effect	*Estimate*	*Std. Error*	*Df*	*t*	*p*
(Intercept)	-9.94	15.15	60	-0.66	0.512
Condition	6.56	15.15	60	0.43	0.665
Age	0.04	0.03	60	1.48	0.139
Object	11.51	8.30	564	1.39	0.165
Block1	9.46	13.75	60	0.69	0.492
Block2	14.30	13.40	60	1.07	0.286
Condition:Age	-0.01	0.03	60	-0.44	0.658
Condition:Object	16.84	8.30	564	2.03	**0.042**
Age:Object	-0.02	0.02	564	-1.40	0.162
Condition:Block1	-1.53	13.75	60	-0.11	0.911
Condition:Block2	-0.93	13.40	60	-0.07	0.944
Age:Block1	-0.01	0.03	60	-0.42	0.677
Age:Block2	-0.03	0.02	60	-1.06	0.288
Object:Block1	-16.65	11.74	564	-1.42	0.156
Object:Block2	2.95	11.74	564	0.25	0.801
Condition:Age:Object	-0.03	0.02	564	-2.10	**0.035**
Condition:Age:Block1	0.00	0.03	60	0.14	0.888
Condition:Age:Block2	0.00	0.02	60	0.07	0.945
Condition:Object:Block1	3.15	11.74	564	0.27	0.788
Condition:Object:Block2	-13.89	11.74	564	-1.18	0.237
Age:Object:Block1	0.03	0.02	564	1.41	0.159
Age:Object:Block2	0.00	0.02	564	-0.18	0.859
Condition:Age:Object:Block1	-0.01	0.02	564	-0.25	0.805
Condition:Age:Object:Block2	0.03	0.02	564	1.18	0.238

Table S4. Stimuli Sentences: Familiarization Phase

Trial	Sentences	
**1**	Wow, a giraffe! The bird should hug him.<br>Ooh, a fish! The pig should hit him.<br>Wow, a chicken! The puppy should cover her.	Hey, a sheep! The bug should kiss him.<br>Hey, a lion! The horse should bump her.<br>Ooh, a butterfly! The tiger should tickle her.
**2**	Wow, a cow! The monkey should cover her.<br>Hey, a bear! The bunny should bump her.<br>Wow, a bee! The dog should hug him.	Ooh, a goose! The frog should tickle her.<br>Ooh, a cat! The elephant should hit him.<br>Hey, a duck! The mouse should kiss him.
**3**	Hey, a pig! The lion should kiss her.<br>Wow, a horse! The giraffe should cover him.<br>Hey, a tiger! The sheep should bump him.	Ooh, a puppy! The fish should tickle him.<br>Ooh, a bird! The butterfly should hit her.<br>Wow, a bug! The chicken should hug her.
**4**	Hey, a frog! The duck should bump him.<br>Wow, a mouse! The cow should hug her.<br>Hey, an elephant! The bear should kiss her.	Ooh, a dog! The goose should hit her.<br>Ooh, a monkey! The cat should tickle him.<br>Wow, a bunny! The bee should cover him.

Table S5. Stimuli Sentences: Test Phase, *Wh*-question Condition*

Trial	Sentences	
**1**	Ooh, which bug should the giraffe tickle?<br>Hey, which puppy should the butterfly kiss?<br>Hey, which bird should the fish bump?	Wow, which tiger should the lion hug?<br>Wow, which pig should the sheep cover?<br>Ooh, which horse should the chicken hit?
**2**	Hey, which dog should the cat bump her?<br>Wow, which elephant should the duck cover her?<br>Wow, which frog should the bear hug him?	Ooh, which bunny should the cow hit him?<br>Hey, which monkey should the goose kiss him?<br>Ooh, which mouse should the bee tickle her?
**3**	Hey, which giraffe should the tiger kiss?<br>Wow, which butterfly should the bug cover?<br>Wow, which fish should the horse hug?	Ooh, which sheep should the puppy hit?<br>Ooh, which lion should the bird tickle?<br>Hey, which chicken should the pig bump?
**4**	Ooh, which duck should the monkey hit her?<br>Hey, which cow should the elephant bump him?<br>Ooh, which bear should the dog tickle him?	Wow, which goose should the mouse cover him?<br>Wow, which cat should the bunny hug her?<br>Hey, which bee should the frog kiss her?
**5**	Wow, which tiger should the fish cover?<br>Ooh, which bug should the lion hit?<br>Ooh, which horse should the sheep tickle?	Hey, which puppy should the giraffe bump?<br>Hey, which bird should the chicken kiss?<br>Wow, which pig should the butterfly hug?
**6**	Wow, which elephant should the goose hug him?<br>Hey, which monkey should the bee bump her?<br>Hey, which dog should the cow kiss him?	Ooh, which bunny should the duck tickle her?<br>Ooh, which mouse should the bear hit him?<br>Wow, which frog should the cat cover her?
**7**	Hey, which dog should the cat bump?<br>Wow, which elephant should the duck cover?<br>Wow, which frog should the bear hug?	Ooh, which bunny should the cow hit?<br>Hey, which monkey should the goose kiss?<br>Ooh, which mouse should the bee tickle?
**8**	Ooh, which bug should the giraffe tickle her?<br>Hey, which puppy should the butterfly kiss him?<br>Hey, which bird should the fish bump her?	Wow, which tiger should the lion hug him?<br>Wow, which pig should the sheep cover her?<br>Ooh, which horse should the chicken hit him?
**9**	Ooh, which duck should the monkey hit?<br>Hey, which cow should the elephant bump?<br>Ooh, which bear should the dog tickle?	Wow, which goose should the mouse cover?<br>Wow, which cat should the bunny hug?<br>Hey, which bee should the frog kiss?
**10**	Hey, which giraffe should the tiger kiss her?<br>Wow, which butterfly should the bug cover him?<br>Wow, which fish should the horse hug her?	Ooh, which sheep should the puppy hit her?<br>Ooh, which lion should the bird tickle him?<br>Hey, which chicken should the pig bump him?
**11**	Wow, which elephant should the goose hug?<br>Hey, which monkey should the bee bump?<br>Hey, which dog should the cow kiss?	Ooh, which bunny should the duck tickle?<br>Ooh, which mouse should the bear hit?<br>Wow, which frog should the cat cover?
**12**	Wow, which tiger should the fish cover her?<br>Ooh, which bug should the lion hit him?<br>Ooh, which horse should the sheep tickle her?	Hey, which puppy should the giraffe bump her?<br>Hey, which bird should the chicken kiss him?<br>Wow, which pig should the butterfly hug him?

*One of four stimulus orders

Table S6. Stimuli Sentences: Test Phase, Declarative Condition*

**Trial**	Sentences	
**1**	Ooh, a bug! The giraffe should tickle.<br>Hey, a puppy! The butterfly should kiss.<br>Hey, a bird! The fish should bump.	Wow, a tiger! The lion should hug.<br>Wow, a pig! The sheep should cover.<br>Ooh, a horse! The chicken should hit.
**2**	Hey, a dog! The cat should bump her.<br>Wow, an elephant! The duck should cover her.<br>Wow, a frog! The bear should hug him.	Ooh, a bunny! The cow should hit him.<br>Hey, a monkey! The goose should kiss him.<br>Ooh, a mouse! The bee should tickle her.
**3**	Hey, a giraffe! The tiger should kiss.<br>Wow, a butterfly! The bug should cover.<br>Wow, a fish! The horse should hug.	Ooh, a sheep! The puppy should hit.<br>Ooh, a lion! The bird should tickle.<br>Hey, a chicken! The pig should bump.
**4**	Ooh, a duck! The monkey should hit her.<br>Hey, a cow! The elephant should bump him.<br>Ooh, a bear! The dog should tickle him.	Wow, a goose! The mouse should cover him.<br>Wow, a cat! The bunny should hug her.<br>Hey, a bee! The frog should kiss her.
**5**	Wow, a tiger! The fish should cover.<br>Ooh, a bug! The lion should hit.<br>Ooh, a horse! The sheep should tickle.	Hey, a puppy! The giraffe should bump.<br>Hey, a bird! The chicken should kiss.<br>Wow, a pig! The butterfly should hug.
**6**	Wow, an elephant! The goose should hug him.<br>Hey, a monkey! The bee should bump her.<br>Hey, a dog! The cow should kiss him.	Ooh, a bunny! The duck should tickle her.<br>Ooh, a mouse! The bear should hit him.<br>Wow, a frog! The cat should cover her.
**7**	Hey, a dog! The cat should bump.<br>Wow, an elephant! The duck should cover.<br>Wow, a frog! The bear should hug.	Ooh, a bunny! The cow should hit.<br>Hey, a monkey! The goose should kiss.<br>Ooh, a mouse! The bee should tickle.
**8**	Ooh, a bug! The giraffe should tickle her.<br>Hey, a puppy! The butterfly should kiss him.<br>Hey, a bird! The fish should bump her.	Wow, a tiger! The lion should hug him.<br>Wow, a pig! The sheep should cover her.<br>Ooh, a horse! The chicken should hit him.
**9**	Ooh, a duck! The monkey should hit.<br>Hey, a cow! The elephant should bump.<br>Ooh, a bear! The dog should tickle.	Wow, a goose! The mouse should cover.<br>Wow, a cat! The bunny should hug.<br>Hey, a bee! The frog should kiss.
**10**	Hey, a giraffe! The tiger should kiss her.<br>Wow, a butterfly! The bug should cover him.<br>Wow, a fish! The horse should hug her.	Ooh, a sheep! The puppy should hit her.<br>Ooh, a lion! The bird should tickle him.<br>Hey, a chicken! The pig should bump him.
**11**	Wow, an elephant! The goose should hug.<br>Hey, a monkey! The bee should bump.<br>Hey, a dog! The cow should kiss.	Ooh, a bunny! The duck should tickle.<br>Ooh, a mouse! The bear should hit.<br>Wow, a frog! The cat should cover.
**12**	Wow, a tiger! The fish should cover her.<br>Ooh, a bug! The lion should hit him.<br>Ooh, a horse! The sheep should tickle her.	Hey, a puppy! The giraffe should bump her.<br>Hey, a bird! The chicken should kiss him.<br>Wow, a pig! The butterfly should hug him.

*One of four stimulus orders

SI References

1. J. Zehr, F. Schwarz, *PennController for Internet Based Experiments (IBEX).* (2018). Available at https://doi.org/10.17605/OSF.IO/MD832
